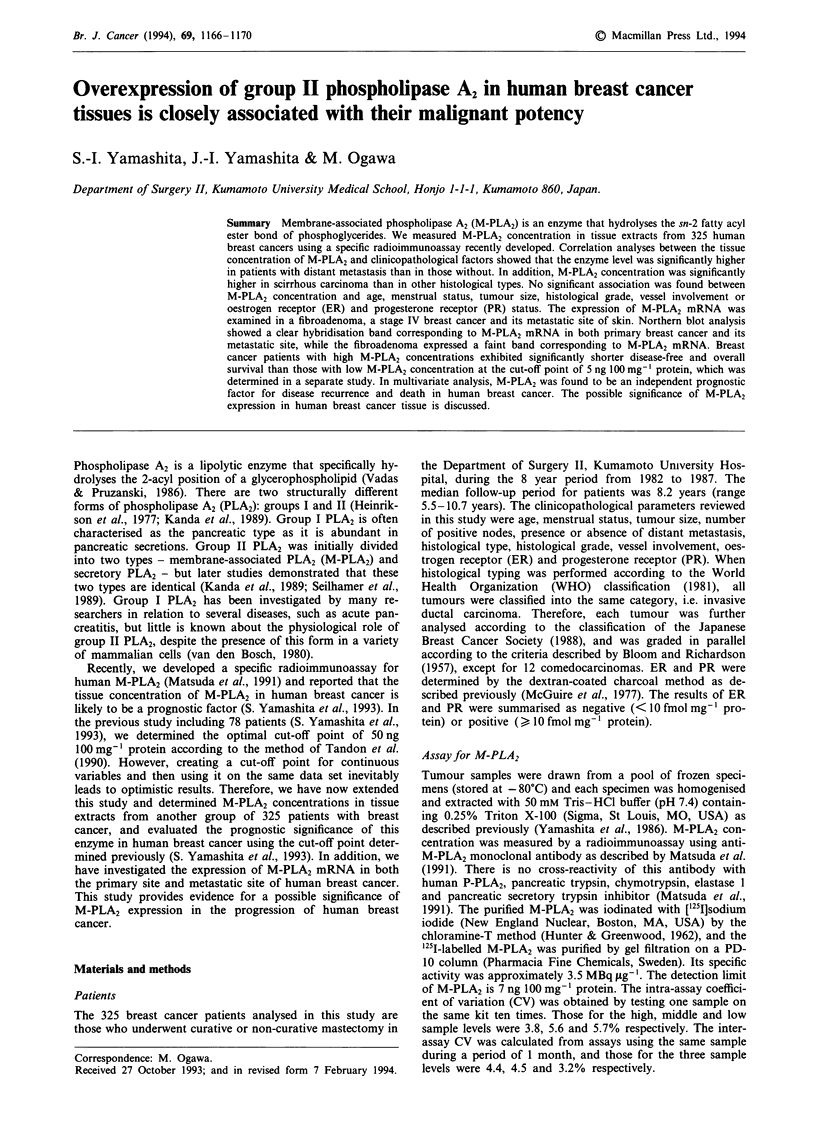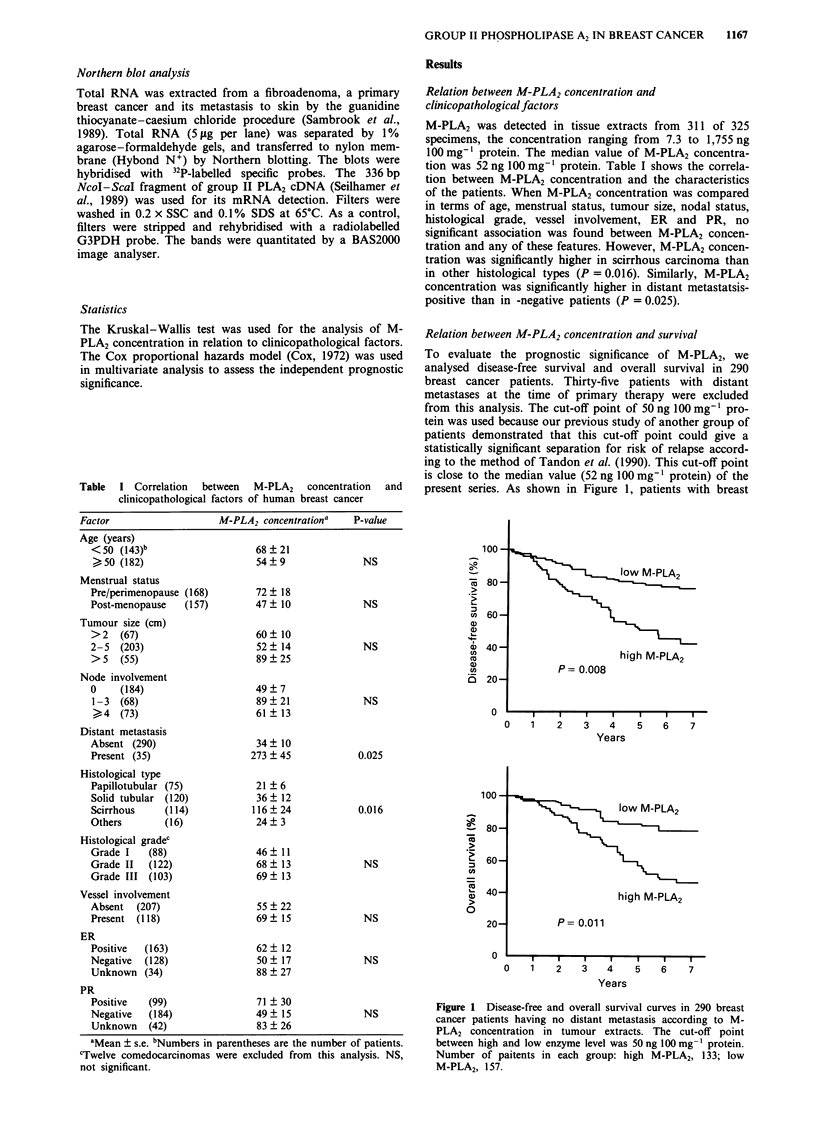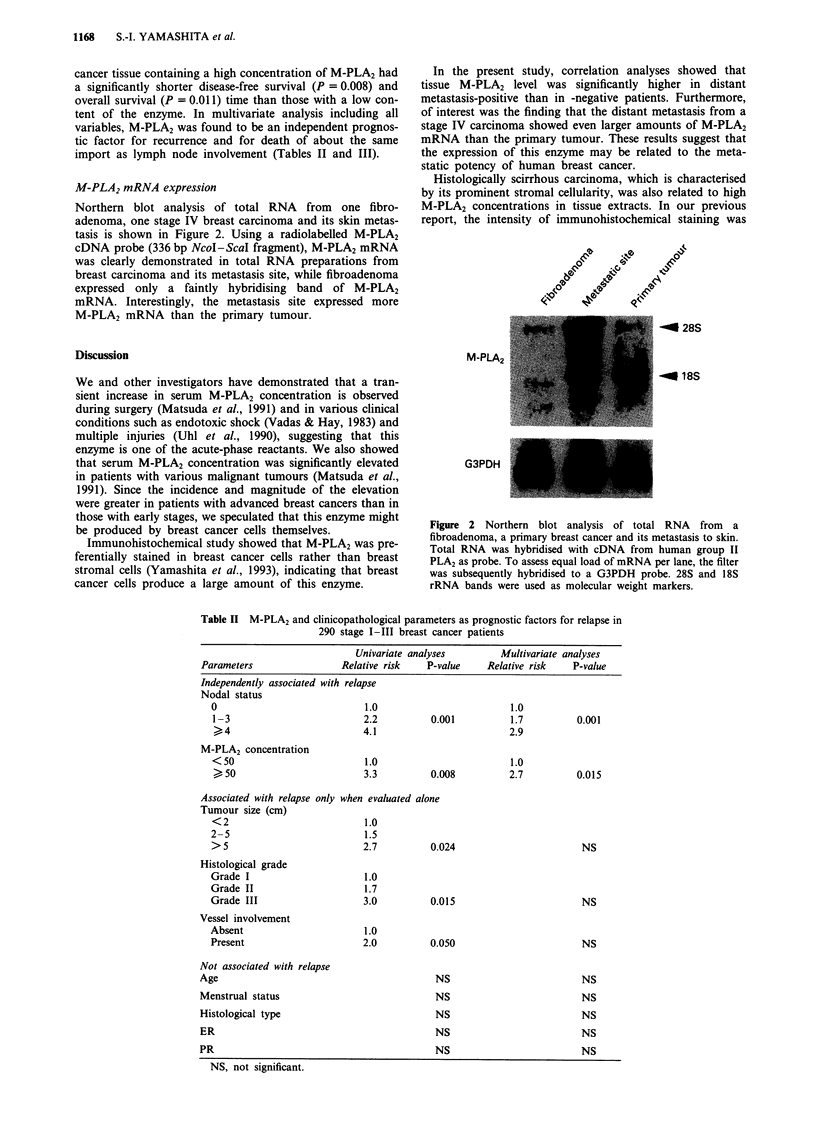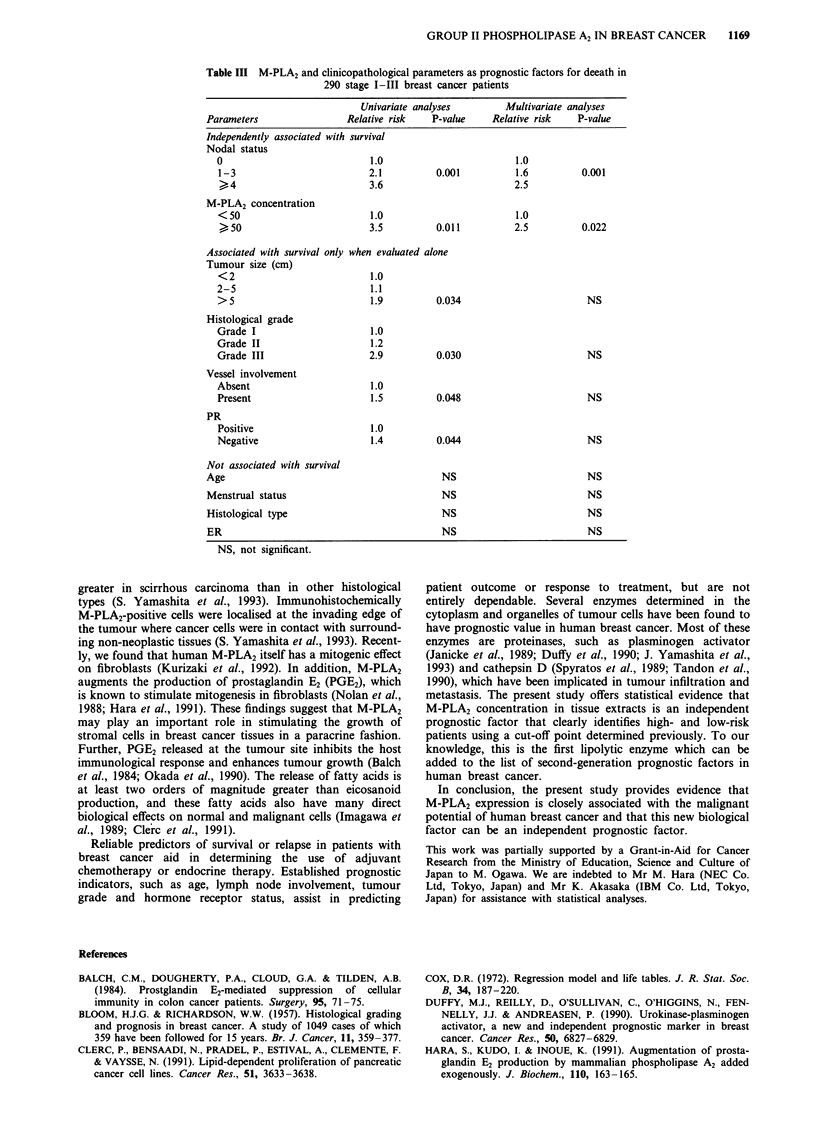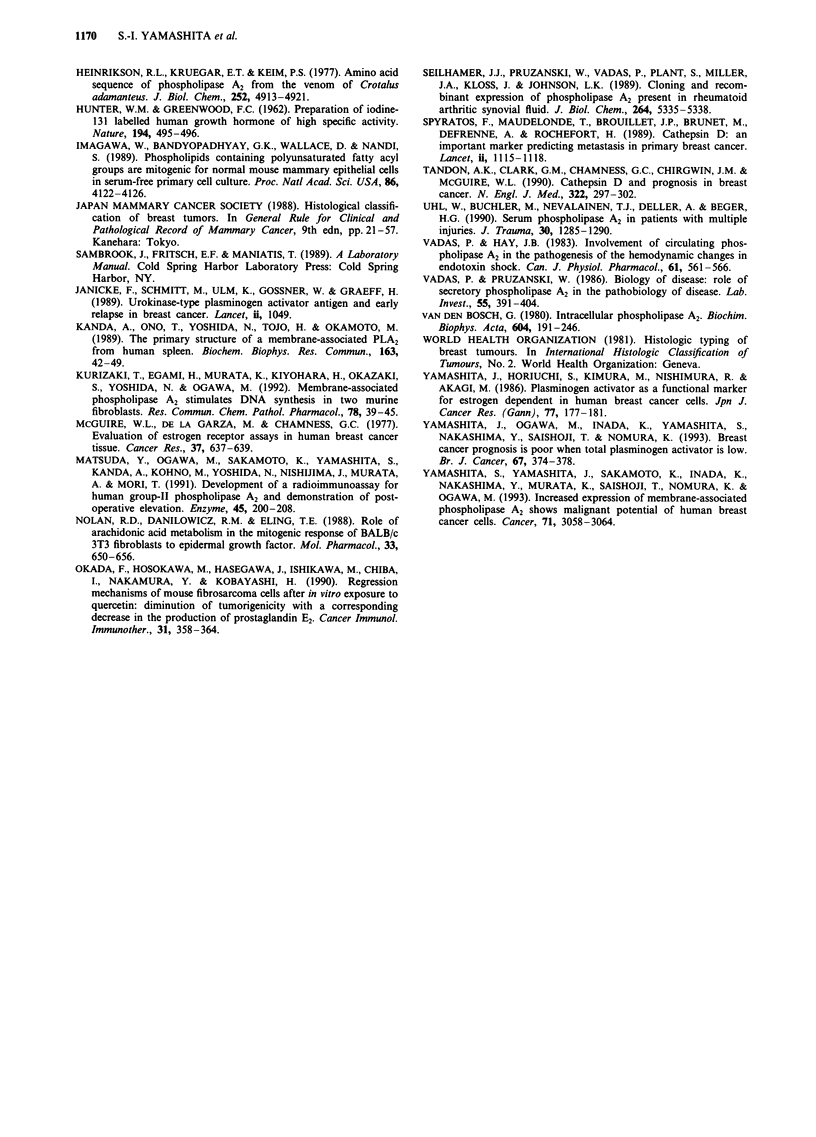# Overexpression of group II phospholipase A2 in human breast cancer tissues is closely associated with their malignant potency.

**DOI:** 10.1038/bjc.1994.229

**Published:** 1994-06

**Authors:** S. Yamashita, J. Yamashita, M. Ogawa

**Affiliations:** Department of Surgery II, Kumamoto University Medical School, Japan.

## Abstract

**Images:**


Br. J. Cancer (1994), 69, 1166–1170 © Macmillan Press Ltd., 1994

### Overexpression of group II phospholipase $A_2$ in human breast cancer tissues is closely associated with their malignant potency

S.-I. Yamashita, J.-I. Yamashita & M. Ogawa

*Department of Surgery II, Kumamoto University Medical School, Honjo 1-1-1, Kumamoto 860, Japan.*

**Summary** Membrane-associated phospholipase $A_2$ (M-$PLA_2$) is an enzyme that hydrolyses the *sn*-2 fatty acyl ester bond of phosphoglycerides. We measured M-$PLA_2$ concentration in tissue extracts from 325 human breast cancers using a specific radioimmunoassay recently developed. Correlation analyses between the tissue concentration of M-$PLA_2$ and clinicopathological factors showed that the enzyme level was significantly higher in patients with distant metastasis than in those without. In addition, M-$PLA_2$ concentration was significantly higher in scirrhous carcinoma than in other histological types. No significant association was found between M-$PLA_2$ concentration and age, menstrual status, tumour size, histological grade, vessel involvement or oestrogen receptor (ER) and progesterone receptor (PR) status. The expression of M-$PLA_2$ mRNA was examined in a fibroadenoma, a stage IV breast cancer and its metastatic site of skin. Northern blot analysis showed a clear hybridisation band corresponding to M-$PLA_2$ mRNA in both primary breast cancer and its metastatic site, while the fibroadenoma expressed a faint band corresponding to M-$PLA_2$ mRNA. Breast cancer patients with high M-$PLA_2$ concentrations exhibited significantly shorter disease-free and overall survival than those with low M-$PLA_2$ concentration at the cut-off point of 5 ng 100 $mg^{-1}$ protein, which was determined in a separate study. In multivariate analysis, M-$PLA_2$ was found to be an independent prognostic factor for disease recurrence and death in human breast cancer. The possible significance of M-$PLA_2$ expression in human breast cancer tissue is discussed.

Phospholipase $A_2$ is a lipolytic enzyme that specifically hydrolyses the 2-acyl position of a glycerophospholipid (Vadas & Pruzanski, 1986). There are two structurally different forms of phospholipase $A_2$ ($PLA_2$): groups I and II (Heinrikson *et al.*, 1977; Kanda *et al.*, 1989). Group I $PLA_2$ is often characterised as the pancreatic type as it is abundant in pancreatic secretions. Group II $PLA_2$ was initially divided into two types – membrane-associated $PLA_2$ (M-$PLA_2$) and secretory $PLA_2$ – but later studies demonstrated that these two types are identical (Kanda *et al.*, 1989; Seilhamer *et al.*, 1989). Group I $PLA_2$ has been investigated by many researchers in relation to several diseases, such as acute pancreatitis, but little is known about the physiological role of group II $PLA_2$, despite the presence of this form in a variety of mammalian cells (van den Bosch, 1980).

Recently, we developed a specific radioimmunoassay for human M-$PLA_2$ (Matsuda *et al.*, 1991) and reported that the tissue concentration of M-$PLA_2$ in human breast cancer is likely to be a prognostic factor (S. Yamashita *et al.*, 1993). In the previous study including 78 patients (S. Yamashita *et al.*, 1993), we determined the optimal cut-off point of 50 ng 100 $mg^{-1}$ protein according to the method of Tandon *et al.* (1990). However, creating a cut-off point for continuous variables and then using it on the same data set inevitably leads to optimistic results. Therefore, we have now extended this study and determined M-$PLA_2$ concentrations in tissue extracts from another group of 325 patients with breast cancer, and evaluated the prognostic significance of this enzyme in human breast cancer using the cut-off point determined previously (S. Yamashita *et al.*, 1993). In addition, we have investigated the expression of M-$PLA_2$ mRNA in both the primary site and metastatic site of human breast cancer. This study provides evidence for a possible significance of M-$PLA_2$ expression in the progression of human breast cancer.

### Materials and methods

### *Patients*

The 325 breast cancer patients analysed in this study are those who underwent curative or non-curative mastectomy in the Department of Surgery II, Kumamoto University Hospital, during the 8 year period from 1982 to 1987. The median follow-up period for patients was 8.2 years (range 5.5–10.7 years). The clinicopathological parameters reviewed in this study were age, menstrual status, tumour size, number of positive nodes, presence or absence of distant metastasis, histological type, histological grade, vessel involvement, oestrogen receptor (ER) and progesterone receptor (PR). When histological typing was performed according to the World Health Organization (WHO) classification (1981), all tumours were classified into the same category, i.e. invasive ductal carcinoma. Therefore, each tumour was further analysed according to the classification of the Japanese Breast Cancer Society (1988), and was graded in parallel according to the criteria described by Bloom and Richardson (1957), except for 12 comedocarcinomas. ER and PR were determined by the dextran-coated charcoal method as described previously (McGuire *et al.*, 1977). The results of ER and PR were summarised as negative ($<$10 fmol $mg^{-1}$ protein) or positive ($\geqslant$10 fmol $mg^{-1}$ protein).

### *Assay for M-$PLA_2$*

Tumour samples were drawn from a pool of frozen specimens (stored at $-80$°C) and each specimen was homogenised and extracted with 50 mM Tris–HCl buffer (pH 7.4) containing 0.25% Triton X-100 (Sigma, St Louis, MO, USA) as described previously (Yamashita *et al.*, 1986). M-$PLA_2$ concentration was measured by a radioimmunoassay using anti-M-$PLA_2$ monoclonal antibody as described by Matsuda *et al.* (1991). There is no cross-reactivity of this antibody with human P-$PLA_2$, pancreatic trypsin, chymotrypsin, elastase 1 and pancreatic secretory trypsin inhibitor (Matsuda *et al.*, 1991). The purified M-$PLA_2$ was iodinated with [$^{125}$I]sodium iodide (New England Nuclear, Boston, MA, USA) by the chloramine-T method (Hunter & Greenwood, 1962), and the $^{125}$I-labelled M-$PLA_2$ was purified by gel filtration on a PD-10 column (Pharmacia Fine Chemicals, Sweden). Its specific activity was approximately 3.5 MBq $\mu g^{-1}$. The detection limit of M-$PLA_2$ is 7 ng 100 $mg^{-1}$ protein. The intra-assay coefficient of variation (CV) was obtained by testing one sample on the same kit ten times. Those for the high, middle and low sample levels were 3.8, 5.6 and 5.7% respectively. The inter-assay CV was calculated from assays using the same sample during a period of 1 month, and those for the three sample levels were 4.4, 4.5 and 3.2% respectively.

Correspondence: M. Ogawa.
Received 27 October 1993; and in revised form 7 February 1994.

*Northern blot analysis*

Total RNA was extracted from a fibroadenoma, a primary breast cancer and its metastasis to skin by the guanidine thiocyanate–caesium chloride procedure (Sambrook *et al.*, 1989). Total RNA (5 μg per lane) was separated by 1% agarose–formaldehyde gels, and transferred to nylon membrane (Hybond $N^+$) by Northern blotting. The blots were hybridised with $^{32}P$-labelled specific probes. The 336 bp *NcoI–ScaI* fragment of group II $PLA_2$ cDNA (Seilhamer *et al.*, 1989) was used for its mRNA detection. Filters were washed in 0.2 × SSC and 0.1% SDS at 65°C. As a control, filters were stripped and rehybridised with a radiolabelled G3PDH probe. The bands were quantitated by a BAS2000 image analyser.

*Statistics*

The Kruskal–Wallis test was used for the analysis of M-$PLA_2$ concentration in relation to clinicopathological factors. The Cox proportional hazards model (Cox, 1972) was used in multivariate analysis to assess the independent prognostic significance.

**Table I** Correlation between M-$PLA_2$ concentration and clinicopathological factors of human breast cancer

| *Factor* | *M-$PLA_2$ concentration*[a] | P-*value* |
|---|---|---|
| Age (years) | | |
| <50 (143)[b] | 68 ± 21 | |
| ≥50 (182) | 54 ± 9 | NS |
| Menstrual status | | |
| Pre/perimenopause (168) | 72 ± 18 | |
| Post-menopause (157) | 47 ± 10 | NS |
| Tumour size (cm) | | |
| >2 (67) | 60 ± 10 | |
| 2-5 (203) | 52 ± 14 | NS |
| >5 (55) | 89 ± 25 | |
| Node involvement | | |
| 0 (184) | 49 ± 7 | |
| 1-3 (68) | 89 ± 21 | NS |
| ≥4 (73) | 61 ± 13 | |
| Distant metastasis | | |
| Absent (290) | 34 ± 10 | |
| Present (35) | 273 ± 45 | 0.025 |
| Histological type | | |
| Papillotubular (75) | 21 ± 6 | |
| Solid tubular (120) | 36 ± 12 | |
| Scirrhous (114) | 116 ± 24 | 0.016 |
| Others (16) | 24 ± 3 | |
| Histological grade[c] | | |
| Grade I (88) | 46 ± 11 | |
| Grade II (122) | 68 ± 13 | NS |
| Grade III (103) | 69 ± 13 | |
| Vessel involvement | | |
| Absent (207) | 55 ± 22 | |
| Present (118) | 69 ± 15 | NS |
| ER | | |
| Positive (163) | 62 ± 12 | |
| Negative (128) | 50 ± 17 | NS |
| Unknown (34) | 88 ± 27 | |
| PR | | |
| Positive (99) | 71 ± 30 | |
| Negative (184) | 49 ± 15 | NS |
| Unknown (42) | 83 ± 26 | |

[a]Mean ± s.e. [b]Numbers in parentheses are the number of patients. [c]Twelve comedocarcinomas were excluded from this analysis. NS, not significant.

**Results**

*Relation between M-$PLA_2$ concentration and clinicopathological factors*

M-$PLA_2$ was detected in tissue extracts from 311 of 325 specimens, the concentration ranging from 7.3 to 1,755 ng 100 $mg^{-1}$ protein. The median value of M-$PLA_2$ concentration was 52 ng 100 $mg^{-1}$ protein. Table I shows the correlation between M-$PLA_2$ concentration and the characteristics of the patients. When M-$PLA_2$ concentration was compared in terms of age, menstrual status, tumour size, nodal status, histological grade, vessel involvement, ER and PR, no significant association was found between M-$PLA_2$ concentration and any of these features. However, M-$PLA_2$ concentration was significantly higher in scirrhous carcinoma than in other histological types ($P = 0.016$). Similarly, M-$PLA_2$ concentration was significantly higher in distant metastatsis-positive than in -negative patients ($P = 0.025$).

*Relation between M-$PLA_2$ concentration and survival*

To evaluate the prognostic significance of M-$PLA_2$, we analysed disease-free survival and overall survival in 290 breast cancer patients. Thirty-five patients with distant metastases at the time of primary therapy were excluded from this analysis. The cut-off point of 50 ng 100 $mg^{-1}$ protein was used because our previous study of another group of patients demonstrated that this cut-off point could give a statistically significant separation for risk of relapse according to the method of Tandon *et al.* (1990). This cut-off point is close to the median value (52 ng 100 $mg^{-1}$ protein) of the present series. As shown in Figure 1, patients with breast

**Figure 1** Disease-free and overall survival curves in 290 breast cancer patients having no distant metastasis according to M-$PLA_2$ concentration in tumour extracts. The cut-off point between high and low enzyme level was 50 ng 100 $mg^{-1}$ protein. Number of paitents in each group: high M-$PLA_2$, 133; low M-$PLA_2$, 157.

cancer tissue containing a high concentration of M-$PLA_2$ had a significantly shorter disease-free survival ($P = 0.008$) and overall survival ($P = 0.011$) time than those with a low content of the enzyme. In multivariate analysis including all variables, M-$PLA_2$ was found to be an independent prognostic factor for recurrence and for death of about the same import as lymph node involvement (Tables II and III).

### *M-$PLA_2$ mRNA expression*

Northern blot analysis of total RNA from one fibroadenoma, one stage IV breast carcinoma and its skin metastasis is shown in Figure 2. Using a radiolabelled M-$PLA_2$ cDNA probe (336 bp *NcoI-ScaI* fragment), M-$PLA_2$ mRNA was clearly demonstrated in total RNA preparations from breast carcinoma and its metastasis site, while fibroadenoma expressed only a faintly hybridising band of M-$PLA_2$ mRNA. Interestingly, the metastasis site expressed more M-$PLA_2$ mRNA than the primary tumour.

### Discussion

We and other investigators have demonstrated that a transient increase in serum M-$PLA_2$ concentration is observed during surgery (Matsuda *et al.*, 1991) and in various clinical conditions such as endotoxic shock (Vadas & Hay, 1983) and multiple injuries (Uhl *et al.*, 1990), suggesting that this enzyme is one of the acute-phase reactants. We also showed that serum M-$PLA_2$ concentration was significantly elevated in patients with various malignant tumours (Matsuda *et al.*, 1991). Since the incidence and magnitude of the elevation were greater in patients with advanced breast cancers than in those with early stages, we speculated that this enzyme might be produced by breast cancer cells themselves.

Immunohistochemical study showed that M-$PLA_2$ was preferentially stained in breast cancer cells rather than breast stromal cells (Yamashita *et al.*, 1993), indicating that breast cancer cells produce a large amount of this enzyme.

In the present study, correlation analyses showed that tissue M-$PLA_2$ level was significantly higher in distant metastasis-positive than in -negative patients. Furthermore, of interest was the finding that the distant metastasis from a stage IV carcinoma showed even larger amounts of M-$PLA_2$ mRNA than the primary tumour. These results suggest that the expression of this enzyme may be related to the metastatic potency of human breast cancer.

Histologically scirrhous carcinoma, which is characterised by its prominent stromal cellularity, was also related to high M-$PLA_2$ concentrations in tissue extracts. In our previous report, the intensity of immunohistochemical staining was

**Figure 2** Northern blot analysis of total RNA from a fibroadenoma, a primary breast cancer and its metastasis to skin. Total RNA was hybridised with cDNA from human group II $PLA_2$ as probe. To assess equal load of mRNA per lane, the filter was subsequently hybridised to a G3PDH probe. 28S and 18S rRNA bands were used as molecular weight markers.

**Table II** M-$PLA_2$ and clinicopathological parameters as prognostic factors for relapse in 290 stage I–III breast cancer patients

| *Parameters* | *Univariate analyses* Relative risk | *Univariate analyses* P-value | *Multivariate analyses* Relative risk | *Multivariate analyses* P-value |
|---|---|---|---|---|
| *Independently associated with relapse* | | | | |
| Nodal status | | | | |
| 0 | 1.0 | | 1.0 | |
| 1–3 | 2.2 | 0.001 | 1.7 | 0.001 |
| ⩾4 | 4.1 | | 2.9 | |
| M-$PLA_2$ concentration | | | | |
| <50 | 1.0 | | 1.0 | |
| ⩾50 | 3.3 | 0.008 | 2.7 | 0.015 |
| *Associated with relapse only when evaluated alone* | | | | |
| Tumour size (cm) | | | | |
| <2 | 1.0 | | | |
| 2–5 | 1.5 | | | |
| >5 | 2.7 | 0.024 | | NS |
| Histological grade | | | | |
| Grade I | 1.0 | | | |
| Grade II | 1.7 | | | |
| Grade III | 3.0 | 0.015 | | NS |
| Vessel involvement | | | | |
| Absent | 1.0 | | | |
| Present | 2.0 | 0.050 | | NS |
| *Not associated with relapse* | | | | |
| Age | | NS | | NS |
| Menstrual status | | NS | | NS |
| Histological type | | NS | | NS |
| ER | | NS | | NS |
| PR | | NS | | NS |

NS, not significant.

**Table III** M-$PLA_2$ and clinicopathological parameters as prognostic factors for deeath in 290 stage I–III breast cancer patients

| | *Univariate analyses* | | *Multivariate analyses* | |
|---|---|---|---|---|
| *Parameters* | *Relative risk* | P-*value* | *Relative risk* | P-*value* |
| *Independently associated with survival* | | | | |
| Nodal status | | | | |
| 0 | 1.0 | | 1.0 | |
| 1–3 | 2.1 | 0.001 | 1.6 | 0.001 |
| $\geqslant 4$ | 3.6 | | 2.5 | |
| M-$PLA_2$ concentration | | | | |
| <50 | 1.0 | | 1.0 | |
| $\geqslant 50$ | 3.5 | 0.011 | 2.5 | 0.022 |
| *Associated with survival only when evaluated alone* | | | | |
| Tumour size (cm) | | | | |
| <2 | 1.0 | | | |
| 2–5 | 1.1 | | | |
| >5 | 1.9 | 0.034 | | NS |
| Histological grade | | | | |
| Grade I | 1.0 | | | |
| Grade II | 1.2 | | | |
| Grade III | 2.9 | 0.030 | | NS |
| Vessel involvement | | | | |
| Absent | 1.0 | | | |
| Present | 1.5 | 0.048 | | NS |
| PR | | | | |
| Positive | 1.0 | | | |
| Negative | 1.4 | 0.044 | | NS |
| *Not associated with survival* | | | | |
| Age | | NS | | NS |
| Menstrual status | | NS | | NS |
| Histological type | | NS | | NS |
| ER | | NS | | NS |

NS, not significant.

greater in scirrhous carcinoma than in other histological types (S. Yamashita *et al.*, 1993). Immunohistochemically M-$PLA_2$-positive cells were localised at the invading edge of the tumour where cancer cells were in contact with surrounding non-neoplastic tissues (S. Yamashita *et al.*, 1993). Recently, we found that human M-$PLA_2$ itself has a mitogenic effect on fibroblasts (Kurizaki *et al.*, 1992). In addition, M-$PLA_2$ augments the production of prostaglandin $E_2$ ($PGE_2$), which is known to stimulate mitogenesis in fibroblasts (Nolan *et al.*, 1988; Hara *et al.*, 1991). These findings suggest that M-$PLA_2$ may play an important role in stimulating the growth of stromal cells in breast cancer tissues in a paracrine fashion. Further, $PGE_2$ released at the tumour site inhibits the host immunological response and enhances tumour growth (Balch *et al.*, 1984; Okada *et al.*, 1990). The release of fatty acids is at least two orders of magnitude greater than eicosanoid production, and these fatty acids also have many direct biological effects on normal and malignant cells (Imagawa *et al.*, 1989; Clerc *et al.*, 1991).

Reliable predictors of survival or relapse in patients with breast cancer aid in determining the use of adjuvant chemotherapy or endocrine therapy. Established prognostic indicators, such as age, lymph node involvement, tumour grade and hormone receptor status, assist in predicting patient outcome or response to treatment, but are not entirely dependable. Several enzymes determined in the cytoplasm and organelles of tumour cells have been found to have prognostic value in human breast cancer. Most of these enzymes are proteinases, such as plasminogen activator (Janicke *et al.*, 1989; Duffy *et al.*, 1990; J. Yamashita *et al.*, 1993) and cathepsin D (Spyratos *et al.*, 1989; Tandon *et al.*, 1990), which have been implicated in tumour infiltration and metastasis. The present study offers statistical evidence that M-$PLA_2$ concentration in tissue extracts is an independent prognostic factor that clearly identifies high- and low-risk patients using a cut-off point determined previously. To our knowledge, this is the first lipolytic enzyme which can be added to the list of second-generation prognostic factors in human breast cancer.

In conclusion, the present study provides evidence that M-$PLA_2$ expression is closely associated with the malignant potential of human breast cancer and that this new biological factor can be an independent prognostic factor.

This work was partially supported by a Grant-in-Aid for Cancer Research from the Ministry of Education, Science and Culture of Japan to M. Ogawa. We are indebted to Mr M. Hara (NEC Co. Ltd, Tokyo, Japan) and Mr K. Akasaka (IBM Co. Ltd, Tokyo, Japan) for assistance with statistical analyses.

### References

BALCH, C.M., DOUGHERTY, P.A., CLOUD, G.A. & TILDEN, A.B. (1984). Prostglandin $E_2$-mediated suppression of cellular immunity in colon cancer patients. *Surgery*, **95**, 71–75.

BLOOM, H.J.G. & RICHARDSON, W.W. (1957). Histological grading and prognosis in breast cancer. A study of 1049 cases of which 359 have been followed for 15 years. *Br. J. Cancer*, **11**, 359–377.

CLERC, P., BENSAADI, N., PRADEL, P., ESTIVAL, A., CLEMENTE, F. & VAYSSE, N. (1991). Lipid-dependent proliferation of pancreatic cancer cell lines. *Cancer Res.*, **51**, 3633–3638.

COX, D.R. (1972). Regression model and life tables. *J. R. Stat. Soc. B*, **34**, 187–220.

DUFFY, M.J., REILLY, D., O'SULLIVAN, C., O'HIGGINS, N., FENNELLY, J.J. & ANDREASEN, P. (1990). Urokinase-plasminogen activator, a new and independent prognostic marker in breast cancer. *Cancer Res.*, **50**, 6827–6829.

HARA, S., KUDO, I. & INOUE, K. (1991). Augmentation of prostaglandin $E_2$ production by mammalian phospholipase $A_2$ added exogenously. *J. Biochem.*, **110**, 163–165.

HEINRIKSON, R.L., KRUEGAR, E.T. & KEIM, P.S. (1977). Amino acid sequence of phospholipase $A_2$ from the venom of *Crotalus adamanteus*. *J. Biol. Chem.*, **252**, 4913–4921.

HUNTER, W.M. & GREENWOOD, F.C. (1962). Preparation of iodine-131 labelled human growth hormone of high specific activity. *Nature*, **194**, 495–496.

IMAGAWA, W., BANDYOPADHYAY, G.K., WALLACE, D. & NANDI, S. (1989). Phospholipids containing polyunsaturated fatty acyl groups are mitogenic for normal mouse mammary epithelial cells in serum-free primary cell culture. *Proc. Natl Acad. Sci. USA*, **86**, 4122–4126.

JAPAN MAMMARY CANCER SOCIETY (1988). Histological classification of breast tumors. In *General Rule for Clinical and Pathological Record of Mammary Cancer*, 9th edn, pp. 21–57. Kanehara: Tokyo.

SAMBROOK, J., FRITSCH, E.F. & MANIATIS, T. (1989). *A Laboratory Manual*. Cold Spring Harbor Laboratory Press: Cold Spring Harbor, NY.

JANICKE, F., SCHMITT, M., ULM, K., GOSSNER, W. & GRAEFF, H. (1989). Urokinase-type plasminogen activator antigen and early relapse in breast cancer. *Lancet*, **ii**, 1049.

KANDA, A., ONO, T., YOSHIDA, N., TOJO, H. & OKAMOTO, M. (1989). The primary structure of a membrane-associated $PLA_2$ from human spleen. *Biochem. Biophys. Res. Commun.*, **163**, 42–49.

KURIZAKI, T., EGAMI, H., MURATA, K., KIYOHARA, H., OKAZAKI, S., YOSHIDA, N. & OGAWA, M. (1992). Membrane-associated phospholipase $A_2$ stimulates DNA synthesis in two murine fibroblasts. *Res. Commun. Chem. Pathol. Pharmacol.*, **78**, 39–45.

MCGUIRE, W.L., DE LA GARZA, M. & CHAMNESS, G.C. (1977). Evaluation of estrogen receptor assays in human breast cancer tissue. *Cancer Res.*, **37**, 637–639.

MATSUDA, Y., OGAWA, M., SAKAMOTO, K., YAMASHITA, S., KANDA, A., KOHNO, M., YOSHIDA, N., NISHIJIMA, J., MURATA, A. & MORI, T. (1991). Development of a radioimmunoassay for human group-II phospholipase $A_2$ and demonstration of post-operative elevation. *Enzyme*, **45**, 200–208.

NOLAN, R.D., DANILOWICZ, R.M. & ELING, T.E. (1988). Role of arachidonic acid metabolism in the mitogenic response of BALB/c 3T3 fibroblasts to epidermal growth factor. *Mol. Pharmacol.*, **33**, 650–656.

OKADA, F., HOSOKAWA, M., HASEGAWA, J., ISHIKAWA, M., CHIBA, I., NAKAMURA, Y. & KOBAYASHI, H. (1990). Regression mechanisms of mouse fibrosarcoma cells after *in vitro* exposure to quercetin: diminution of tumorigenicity with a corresponding decrease in the production of prostaglandin $E_2$. *Cancer Immunol. Immunother.*, **31**, 358–364.

SEILHAMER, J.J., PRUZANSKI, W., VADAS, P., PLANT, S., MILLER, J.A., KLOSS, J. & JOHNSON, L.K. (1989). Cloning and recombinant expression of phospholipase $A_2$ present in rheumatoid arthritic synovial fluid. *J. Biol. Chem.*, **264**, 5335–5338.

SPYRATOS, F., MAUDELONDE, T., BROUILLET, J.P., BRUNET, M., DEFRENNE, A. & ROCHEFORT, H. (1989). Cathepsin D: an important marker predicting metastasis in primary breast cancer. *Lancet*, **ii**, 1115–1118.

TANDON, A.K., CLARK, G.M., CHAMNESS, G.C., CHIRGWIN, J.M. & MCGUIRE, W.L. (1990). Cathepsin D and prognosis in breast cancer. *N. Engl. J. Med.*, **322**, 297–302.

UHL, W., BUCHLER, M., NEVALAINEN, T.J., DELLER, A. & BEGER, H.G. (1990). Serum phospholipase $A_2$ in patients with multiple injuries. *J. Trauma*, **30**, 1285–1290.

VADAS, P. & HAY, J.B. (1983). Involvement of circulating phospholipase $A_2$ in the pathogenesis of the hemodynamic changes in endotoxin shock. *Can. J. Physiol. Pharmacol.*, **61**, 561–566.

VADAS, P. & PRUZANSKI, W. (1986). Biology of disease: role of secretory phospholipase $A_2$ in the pathobiology of disease. *Lab. Invest.*, **55**, 391–404.

VAN DEN BOSCH, G. (1980). Intracellular phospholipase $A_2$. *Biochim. Biophys. Acta*, **604**, 191–246.

WORLD HEALTH ORGANIZATION (1981). Histologic typing of breast tumours. In *International Histologic Classification of Tumours*, No. 2. World Health Organization: Geneva.

YAMASHITA, J., HORIUCHI, S., KIMURA, M., NISHIMURA, R. & AKAGI, M. (1986). Plasminogen activator as a functional marker for estrogen dependent in human breast cancer cells. *Jpn J. Cancer Res. (Gann)*, **77**, 177–181.

YAMASHITA, J., OGAWA, M., INADA, K., YAMASHITA, S., NAKASHIMA, Y., SAISHOJI, T. & NOMURA, K. (1993). Breast cancer prognosis is poor when total plasminogen activator is low. *Br. J. Cancer*, **67**, 374–378.

YAMASHITA, S., YAMASHITA, J., SAKAMOTO, K., INADA, K., NAKASHIMA, Y., MURATA, K., SAISHOJI, T., NOMURA, K. & OGAWA, M. (1993). Increased expression of membrane-associated phospholipase $A_2$ shows malignant potential of human breast cancer cells. *Cancer*, **71**, 3058–3064.